# T cell-lymphoma in the eyelid of a 9-year-old English Setter

**DOI:** 10.1186/s13028-018-0432-2

**Published:** 2018-12-06

**Authors:** Lauge Hjorth Mikkelsen, Frederik Holm, Erik Clasen-Linde, Pernille Engraff, Steffen Heegaard

**Affiliations:** 1 Eye Pathology Section, Department of Pathology, Rigshospitalet, Faculty of Health and Medical Sciences, University of Copenhagen, Frederik V’s Vej, 11, 1st Floor, 2100 Copenhagen Ø, Denmark; 2 Department of Ophthalmology, Rigshospitalet, Faculty of Health and Medical Sciences, University of Copenhagen, Copenhagen, Denmark; 3 Anicura, Københavns Dyrehospital, Copenhagen, Denmark

**Keywords:** Canine, Dog, Eyelid, Lymphoma, Neoplasm, T-cell

## Abstract

**Background:**

Eyelid tumours are frequently found in dogs, most of these being benign. In case of an ulcerating eyelid tumour, malignancy must be considered. We report a unique case of a low-grade peripheral T-cell lymphoma in the eyelid of a 9-year-old English Setter.

**Case presentation:**

A 9-year-old Setter presented with a 6-month history of an eyelid ulcer. A malignant eyelid neoplasm was suspected, and the lesion was surgically excised. No other treatment was applied, and 19 months after excision the dog was still well. Histopathology revealed a diffuse lymphocytic infiltrate in the eyelid skin. Ulceration of the epithelium was seen, and the underlying tumour was composed of round and poorly demarcated pleomorphic tumour cells. The cytoplasm was pale and the nuclei heterogeneous. Numerous mitoses were present. The tumour cells stained strongly for CD3. The final diagnosis was a peripheral T-cell lymphoma not otherwise specified (NOS).

**Conclusions:**

This is the first described case of a solitary T-cell lymphoma NOS in the haired eyelid skin in a dog. Lymphoma should be considered in case of a persistent eyelid ulcer and a biopsy should be performed. T-cell lymphoma is generally an aggressive disease; however, indolent cases are well known, and as this case shows, complete excision of a solitary T-cell lymphoma can be curable. Canine cutaneous epitheliotropic T-cell lymphoma is an important differential diagnosis, which must be recognized as the prognosis is very poor and systemic treatment is mandatory. The sub-classification of canine lymphoma is not complete, and further studies are needed to identify lymphoma subgroups and provide treatment guidelines.

## Background

Lymphoma is the most frequent haematologic malignancy among dogs, and the annual incidence rate is 13–114/100,000 [1]. Canine lymphoma may be divided into Hodgkin’s-like and non-Hodgkin’s-like lymphoma, the latter being the most frequent [2]. Furthermore, non-Hodgkin’s-like lymphoma may be subdivided according to the origin of the neoplastic cells [2]. Non-Hodgkin’s-like lymphomas with commitment to the T-cell-lineage represent 20% of all dog lymphomas, while the rest are of B-cell origin [3]. Clinically, lymphadenopathy is the most frequent presentation of lymphoma in dogs, however, extranodal lymphoma may also present with a rapidly growing mass due to accumulation of tumour cells in extranodal tissue [1, 2]. Additionally, in case of systemic disease, symptoms such as fever and weight loss can be present [1, 2].

Histopathologically, most lymphomas are characterized by a diffuse monoclonal lymphocytic infiltrate, however, follicular types also occur [2]. The tumour cells are further characterized based on the growth pattern, nuclear size, nuclear morphology, mitotic index, and immunophaenotype [2].

B- and T-cell lymphomas may be distinguished by immunohistochemistry. In humans, multiple immunohistochemical markers are used to separate lymphoma subtypes; however, many of these markers do not function when applied to canine tissue because most standard antibodies are developed for use in human pathology. In general, tumour cells of T-cell origin demonstrate positivity for cluster of differentiation (CD)3, CD4, and CD8, while tumour cells of B-cell origin are positive when staining for CD19, CD20, CD79α, and PAX-5 [4]. In our experience, the most reliable immunohistochemical stainings for determining the lineage in dog lymphomas are PAX-5 and CD3.

Periocular and intraocular lymphomas are rare in dogs [4–15]. Previously, a T-cell lymphoma in the palpebral conjunctiva of a dog has been reported [9], and to the best of our knowledge, this present case is the first case of a canine solitary cutaneous eyelid T-cell lymphoma.

### Case presentation

**Fig. 1:**
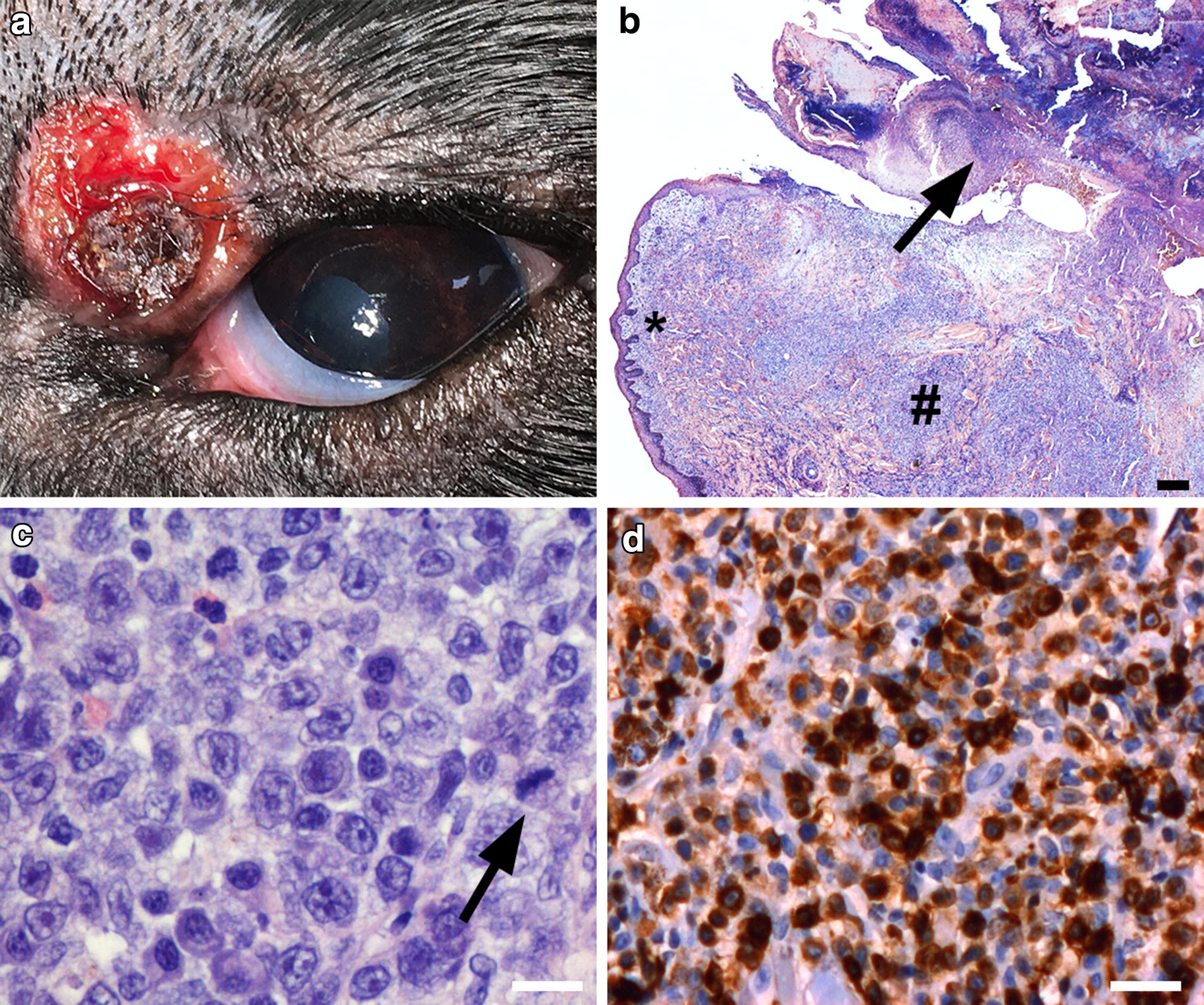
**a** A 9-year-old male English Setter presented with a 6-month history of an eyelid ulcer on top of a tumour measuring 10 × 14 mm. The lesion is round and surrounds a central area of normal eyelid epithelium with hair. **b** In overview, a diffuse infiltrate of lymphoid tumour cells is seen within the epidermis and dermis (#). A large ulceration of the epithelium is seen (arrow). The tumour cells fill the rete ridges (*) and invasion of the epithelium is seen in some areas. Invasion of the adnexal epithelium does not occur (haematoxylin and eosin (HE); bar: 100 µm). **c** The tumour cells have pale cytoplasm and prominent nucleoli. Numerous mitotic figures are seen (arrow). (HE; bar: 30 µm). **d** The tumour cells stain positive in anti-CD3 staining, indicating that the tumour cells are of T-cell origin (bar: 50 µm)

A 9-year-old male English Setter presented with a 6-month history of ulceration of the left upper eyelid as the only clinical finding (Fig. 1a). On examination, the lesion was round, elevated, and well circumscribed. A malignant eyelid tumour was suspected, and the lesion was excised for histopathological examination. A fine-needle aspirate was obtained from a popliteal lymph node from the left side. The popliteal lymph node was chosen, as the owner did not want any expensive diagnostic procedures and the popliteal lymph node was the easiest accessible. The dog was doing well and presented without fever, clinical signs of anaemia, or weight loss. A routine blood profile was unremarkable except for a haematocrit (packed cell volume, PCV) of 37%, which is borderline low. The blood test also showed a serum calcium: 2.43 mmol/L (ref 1.98–3.0 mmol/L), serum albumin: 31 g/L (ref 23–40 g/L), serum alkaline phosphatase: 53 U/L (ref 23–213 U/L), and serum creatinine: 91 µmol/L (ref 44–159 µmol/L). Serum sodium, potassium, chloride, and glucose were also within normal limits. C-reactive protein (CRP) was < 10 mg/L, which is also normal. The owner did not want additional examinations or treatments other than excision of the tumour and for this reason, a thoracic radiograph, abdominal ultrasound, and a full haematological profile were not performed. No systemic treatment was initiated, and 2 months after surgery an unremarkable full haematological profile was obtained. 19 months after the diagnosis the dog was still alive and doing well with no signs of relapse.

The specimen was fixed in 10% neutral buffered formalin immediately after surgical excision, processed and embedded in paraffin. Sections (4 µm) were prepared and stained with haematoxylin and eosin according to standard protocols. Furthermore, sections were routinely stained with periodic acid-Schiff (PAS), Gram stain, and Gomori’s methenamine silver stain (GMS) to detect microorganisms. Giemsa staining was performed to visualize the blood derived cells in the specimen.

Immunohistochemical stainings were performed on a Ventana Benchmark Ultra Platform (Ventana Medical Systems, Tucson, AZ, USA) according to manufacturer’s instructions. The following antibodies were used: CD3 [clone 2GV6, code 790-4341, rabbit anti-human, ready-to-use (RTU), Roche (Hvidovre, Denmark)], PAX-5 [clone SP34, code 790-4420, rabbit anti-human, RTU, Roche (Hvidovre, Denmark)], vimentin [clone V9, code 790-2917, mouse anti-human, RTU, Roche (Hvidovre, Denmark)], and Ki-67 (clone MIB-1, code M724001, mouse anti-human, 1:100, Dako). Positive and negative controls were performed according to the manufacturers’ instructions.

## Results

Microscopically, an ulceration of the eyelid epithelium was seen. A diffuse lymphocytic infiltrate was observed within the epithelium and in the underlying dermis (Fig. 1b). In some areas of the dermis, the adnexal structures were surrounded by tumour cells, however, several unaffected hair follicles and sebaceous glands were also observed. No invasion of the adnexal structures was seen. The excision margins were free from tumour cells. The tumour cells were round, pleomorphic and poorly demarcated. The tumour cells varied in size being approximately 14 µm in diameter and with abundant cytoplasm and large nuclei (Fig. 1c). The nucleoli were prominent and numerous mitotic figures were present. No tingible body macrophages were seen in the specimen. The tumour cells stained light blue with Giemsa stain, in contrast to normal lymphocytes which were more hyperchromatic. Scattered mast cells were seen within the tumour infiltrate, and numerous eosinophils were observed in the area under the ulceration. Numerous Gram-positive cocci were seen in the outer part of the ulceration.

Immunohistochemistry demonstrated membranous and cytoplasmic positivity for CD3 in the tumour cells (Fig. 1d). No reaction was seen for the pan-B-cell marker PAX-5. Staining with Ki-67/MIB1 was positive in approximately 30% of the tumour cells, indicating a rather high mitotic index. No tumour cells were found in the fine-needle aspiration of the popliteal lymph node. The sample was considered diagnostic and a few normal scattered lymphocytes were seen in the specimen. Overall, these findings were consistent with a malignant solitary extranodal T-cell-lymphoma not otherwise specified (NOS) [1, 3].

## Discussion and conclusions

This case describes the first case of a solitary T-cell lymphoma in the hairy skin of the eyelid in a dog presenting with a cutaneous ulceration in the eyelid. Previously, a case of a conjunctival T-cell lymphoma has been reported [9]. The conjunctiva is a mucosal membrane which harbours a part of the innate immune system known as mucosa associated lymphoid tissue (MALT). For this reason, the immunology and biology of conjunctival lymphomas is different from eyelid skin lymphoma. Although very rare, lymphoma should be considered in case of a persistent treatment refractory eyelid ulcer, and a biopsy should always be performed. Other, more frequently occurring differential diagnoses such as immune mediated blepharitis, chalazion, hordeolum, insect bites, infections, trauma, histiocytoma, mast cell tumours, or benign tumours such as sebaceous adenoma should be ruled out.

In most cases of canine lymphoma, histopathology and relatively simple immunophaenotyping is sufficient for obtaining the correct diagnosis [1, 3]. In the present case, the lymphocytic tumour cells were round and varied in size. Immunohistochemistry demonstrated tumour cells of T-cell origin. Overall, these findings are suggestible of a peripheral T-cell lymphoma NOS, according to the WHO classification of canine lymphomas [1, 3]. This type of lymphoma represents approximately 16% of all canine lymphomas, however, it has never been reported in the eyelid [1]. The presence of bacteria within the specimen was interpreted as a secondary infection.

Diagnostic imaging is not recommended for staging purposes on a routine basis [1], and in our case it was not performed due to the owners’ wish. However, diagnostic imaging may be considered on an individual basis [1]. In cases with clinically suspected systemic disease, a blood test and/or diagnostic imaging can be performed to carry out a rough staging of the lymphoma according to the WHO stages for canine multicentric lymphoma [1]. For a full staging, a bone marrow biopsy should also be obtained. Staging may, however, not be necessary in all cases of a solitary lymphoma and in the present case it was not performed. If staging is performed, the stage is not necessarily a predictor of better survival [1, 13]. In general, however, stage I-II disease has a favourable prognosis, whereas stage V lymphoma usually has a poor prognosis if extensive bone marrow involvement is present [16].

Clinically, no lymph nodes were involved in the present case, and the blood tests were normal. Serum calcium and albumin levels may be elevated in case of a disseminated lymphoma, and in some studies, low albumin concentration has been associated with a poor prognosis [17]. In the present case, both serum calcium and albumin levels were normal. Additionally, in the present case we examined a popliteal lymph node due to the owners wish. If a clinically enlarged lymph node is found, this lymph node should be excised and histopathologically examined. In cases where no clinically enlarged lymph node is present, a regional lymph node should be preferred to evaluate systemic involvement.

Overall, the present case is most likely a stage Ia disease, which corresponds to a lymphoma restricted to a single node or lymphoid tissue in a single organ with absence of systemic signs. No detailed haematology, thoracic radiographs, or abdominal ultrasound was performed prior to surgery, and this makes our staging questionable. However, because the dog is doing well with no signs of progression 19 months after excision with free margins, a low-grade lymphoma seems plausible.

An important differential diagnosis of a T-cell lymphoma in the haired skin of a dog is the so-called canine cutaneous epitheliotropic T-cell lymphoma (CETL) [16]. This diagnosis was considered in the present case mainly due to the epitheliotropic growth of the lesion and the ulceration of the overlying epidermis. On the other hand, only few adnexal structures were surrounded by tumour cells, and invasion of tumour cells into the adnexal structures was not observed. Furthermore, dogs with CETL often have multiple lesions and systemic symptoms, which was not the case in the present study [18].

In general, lymphoma remains one of the most chemotherapy-responsive cancers in dogs [1, 19]. Unfortunately, peripheral T-cell lymphoma NOS is in most cases an unpredictable and aggressive haematologic cancer with a poor prognosis, and the mean survival time is approximately 6 months [1]. The poor prognosis is possibly due to the aggressive nature of the disease, histologically demonstrated by a high proliferation index and a high degree of cellular pleomorphism. The recommended treatment is high-intensity CHOP (cyclophosphamide, hydroxydaunorubicin, oncovin [vincristine], and prednisone)-based protocols combined with various rescue protocols (such as mechlorethamine/procarbazine-based) [1, 3, 19]. Some authors suggest that l-asparaginase-MOPP (mustargen, oncovin, procarbazine, prednisone) may be superior to standard CHOP-protocols in canine T-cell lymphomas [1]. Other studies suggest using protocols based on an alkylating agent, lomustine, in the treatment of canine T-cell lymphomas [20]. However, indolent cases of low-stage solitary peripheral T-cell lymphoma in dogs may not require systemic treatment after successful excision of the tumour [19]. In this light, it seems that the inclusion of these low-grade cases in the aggressive group of peripheral T-cell lymphoma NOS is not adequate, and some dogs will receive aggressive chemotherapy, which is not needed and an expensive solution. In humans, lymphoma may be sub-classified according to several specific genetic abnormalities; however, in animals, the information on molecular biology is still very limited [1]. Nonetheless, it seems that canine lymphomas carry less genomic instabilities compared to human lymphomas [1]. This is an interesting point, as other mechanisms than genomic instabilities are possibly involved in canine lymphoma pathogenesis when compared to human lymphoma, and thus canine lymphoma should possibly be sub-classified according to other features than lymphomas of humans.

This is the first reported case of a cutaneous T-cell-lymphoma in the eyelid of a dog, a so-called peripheral T-cell lymphoma NOS with an indolent course. The existing classification systems of canine T-cell lymphomas may not be sufficient and there may exist multiple sub-classifications of T-cell lymphoma in dogs. Studies aiming to sub-classify canine lymphoma based on detailed immunophaenotyping and molecular biology have not been made. It would be of interest to perform larger multicentre studies to divide canine T-cell lymphomas into specific subcategories with differing prognoses and sensitivities to various treatment regimens.
